# Venezuelan Equine Encephalitis Viruses (VEEV) in Argentina: Serological Evidence of Human Infection

**DOI:** 10.1371/journal.pntd.0002551

**Published:** 2013-12-12

**Authors:** María Belén Pisano, Griselda Oria, Geraldine Beskow, Javier Aguilar, Brenda Konigheim, María Luisa Cacace, Luis Aguirre, Marina Stein, Marta Silvia Contigiani

**Affiliations:** 1 Instituto de Virología “Dr. J. M. Vanella”, Facultad de Ciencias Médicas, Universidad Nacional de Córdoba, Enfermera Gordillo Gómez s/n, Ciudad Universitaria, Córdoba, Argentina; 2 Instituto de Medicina Regional, Universidad Nacional del Nordeste, Resistencia, Chaco, Argentina; 3 Hospital San Vicente de Paul, Orán, Salta, Argentina; 4 Hospital Dante Tardelli, Pampa del Indio, Chaco, Argentina; University of Texas Medical Branch, United States of America

## Abstract

Venezuelan equine encephalitis viruses (VEEV) are responsible for human diseases in the Americas, producing severe or mild illness with symptoms indistinguishable from dengue and other arboviral diseases. For this reason, many cases remain without certain diagnosis. Seroprevalence studies for VEEV subtypes IAB, ID, IF (Mosso das Pedras virus; MDPV), IV (Pixuna virus; PIXV) and VI (Rio Negro virus; RNV) were conducted in persons from Northern provinces of Argentina: Salta, Chaco and Corrientes, using plaque reduction neutralization test (PRNT). RNV was detected in all studied provinces. Chaco presented the highest prevalence of this virus (14.1%). Antibodies against VEEV IAB and -for the first time- against MDPV and PIXV were also detected in Chaco province. In Corrientes, seroprevalence against RNV was 1.3% in the pediatric population, indicating recent infections. In Salta, this was the first investigation of VEEV members, and antibodies against RNV and PIXV were detected. These results provide evidence of circulation of many VEE viruses in Northern Argentina, showing that surveillance of these infectious agents should be intensified.

## Author Summary

Venezuelan equine encephalitis viruses (VEEV) are responsible for human diseases in the Americas. They produce severe or mild illnesses with symptoms indistinguishable from dengue and other arboviral diseases; for this reason, many cases remain undiagnosed. We detected neutralizing antibodies (NTAbs) against VEEV IAB, VEEV ID, MDPV (VEEV subtype IF), PIXV (VEEV subtype IV) and RNV (VEEV subtype VI) in human serum samples of Northern provinces of Argentina. Chaco province showed presence of NTAbs against VEEV IAB, MDPV, PIXV and RNV. In Corrientes province, we detected NTAbs against RNV in a pediatric population. NTAbs against PIXV and RNV were also detected in Salta province. These findings demonstrated the circulation of many VEEV strains in Northern Argentina and underscore the need for surveillance of dengue like illness in this region.

## Introduction

Venezuelan equine encephalitis (VEE) is a reemerging mosquito-borne viral disease that is severely debilitating and sometimes fatal to humans [1]. The etiological agent, VEE virus (VEEV), belongs to the VEE complex (*Togaviridae: Alphavirus*), one of the major alphavirus serogroups found in the New World [2]. Members of the VEE complex are distributed throughout America and have been originally classified in subtypes based on their serology; however, they are now considered different virus species. Only subtypes IAB and IC are considered epidemic/epizootic varieties since they have been responsible for outbreaks involving equine and human cases [3]. These subtypes undergo an amplification cycle that involves equids, which develop high titer viremia, and mosquitoes [1]. Enzootic strains (subtype I varieties ID, IE, IF and subtypes II to VI) are not associated with equine disease, producing low titer viremia, with the exception of VEEV IE. Interestingly, strains in this subtype have been responsible for epizootics in Mexico and appear to be equine neurovirulent, but are not known to produce high titer viremia in equids [4]. Enzootic strains circulate in forested or swamp habitats, where rodents serve as reservoir hosts and *Culex* mosquitoes -mainly in the subgenus *Melanoconion*- act as vectors [5]. However, these viruses have also been detected in urban areas [6], [7], [8]. Human infection by any of these strains can be completely asymptomatic or present with a mild disease, with symptoms similar to dengue or influenza, although a fatal human case caused by enzootic VEEV ID was reported in Panama in 1961 [1]. Enzootic VEEV are increasingly recognized as important endemic pathogens of people who live near the enzootic transmission foci and/or enter the habitats where enzootic circulation occurs [1]. Some of these enzootic viruses are postulated to be progenitors of epizootic strains [4].

In Argentina, the circulation of Rio Negro Virus (VEEV subtype VI; RNV) is well known; it was isolated for the first time in 1980 by Mitchell et al. from mosquitoes of Chaco province [9]. In 1989, Contigiani et al. reported an outbreak of acute febrile illness in humans from General Belgrano Island (Formosa province) associated to RNV, with symptoms indistinguishable from dengue [10]. Subsequent serological studies carried out in the same area showed the presence of human antibodies not only against RNV, but also against subtype IAB (TC83 vaccine strain) [11]. Recent investigations have reported the molecular detection of RNV and Pixuna Virus (VEEV subtype IV; PIXV) in Chaco and Tucumán provinces [7], [8], demonstrating that more than one VEEV is currently active in Argentina. RNV has also been detected in Córdoba Province [12].

Because epidemics of arboviruses often receive notice only when they are acute and massive, the public loses sight of ongoing transmission, which has a significant daily impact on the life of people living in endemic countries. These diseases are often ignored and neglected because they have not yet impacted the lives of those living in affluent areas. They are understudied and go unnoticed until outbreaks occur [13]. The epidemiological characteristics and geographic range for many endemic arboviruses in South America are poorly understood [14]. This is the case for endemic VEE, which is underreported in many parts of the continent, where enzootic circulation occurs and surveillance of febrile illness is limited, such as in Argentina. To begin to address this gap, we proposed to determine the occurrence of VEEV infection in humans of the North part of the country -where circulation of RNV and PIXV is well known-, and investigated the presence of neutralizing antibodies (NTAbs) against VEEV IAB, VEEV ID, Mosso das Pedras virus (VEEV subtype IF; MDPV), PIXV and RNV in human sera obtained during the period 2006–2011.

## Materials and Methods

### Ethics statement

This study was designed as a non-associated, anonymous survey: data registered were only number of sample, date of sampling, age of the patient (years), gender and address (street and neighborhood). It was approved by the ethics committee of the Faculty of Medicine, National University of Northeast (UNNE) and conducted within the project N°FBBI11/10.

### Sites and samples analyzed

**Figure 1:**
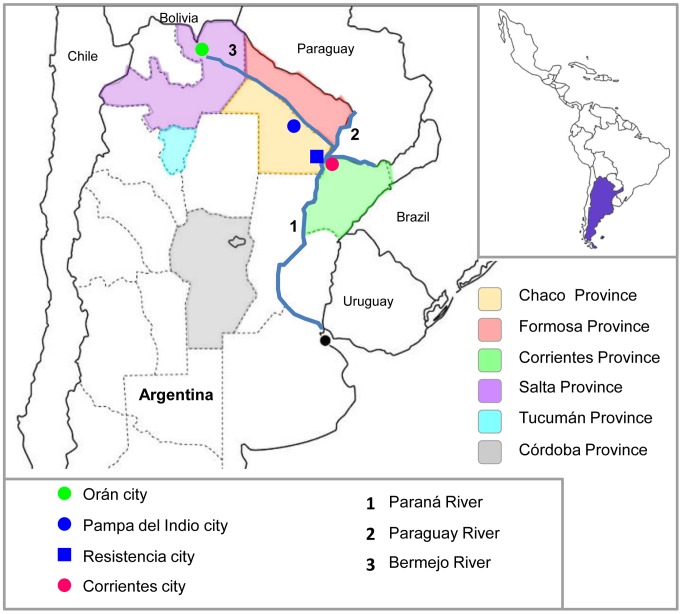
Map of Argentina. Provinces evaluated in this study and provinces where VEEVs have previously been detected.

All the studied locations are indicated in Figure 1. The sera were obtained from people without symptoms who attended health centers to perform routine or other analysis within the project “Ecoepidemiology of arboviruses in Argentina”.

#### Chaco Province

we studied 294 serum samples of people aged between 2 and 88 years: 172 obtained in the Central Laboratory of Chaco Province, located in the capital city of Resistencia (27°27′76″S; 58°59′79″W), from March to July 2007; and 122 from Pampa del Indio (26°02′54″S; 59°56′32″W), obtained in the Dante Tardelli Hospital between December 2010 and May 2011.

Chaco Province is characterized by a very large plain with smooth slopes towards the southeast. The climate is subtropical with a dry season. The eastern woodland of the province, where this study was conducted, is within a flood plain with imperfect or poor drainage. The soil permeability is moderately slow, which allows the formation of flooding areas [15]. The mean annual temperature is 23°C and the mean rainfall is 1280 mm. The rainy season is between November–April, with peaks of rainfall in spring (September–October) and autumn (March–April) [15]. Resistencia is the capital city of the Province, located at the banks of Paraná River (Figure 1). Pampa del Indio is a location near the Provincial Park of the same name (30 km far), in which is the Bermejo River (Figure 1).

#### Corrientes Province

149 serum samples from individuals aged 0 to 20 years were investigated. These samples were obtained in the Juan Pablo II Pediatric Hospital, located in Corrientes city (27°28′16″S; 58°50′22″W), capital of the province, from December 2006 to April 2007.

In Corrientes, three phytogeographical zones converge. Due to its location, there are three climatic zones: humid subtropical in the Northeast of the province, subtropical with dry season towards the middle Paraná and temperate with rainfall throughout the year in the Southern. The mean annual temperature is 21°C and the mean rainfall is 1450 mm [16]. The city of Corrientes is located in the other side of the Paraná River, in front of Resistencia city (Figure 1).

#### Salta Province

195 human serum samples aged 1 to 78 years were studied. They were obtained in the San Vicente de Paul Hospital, from Orán city (23°08′19″S; 64°19′06″W), during 2009.

Salta Province has an extremely varied relief, from the peaks of Los Andes to the plains of Chaco, which determines the presence of different types of climate and well contrasted environments. The mean annual temperature is 18°C and the mean rainfall is 400 mm. In Orán, the climate is subtropical of the highlands, with a mean temperature of 22°C [17].

### Plaque reduction neutralization test (PRNT)

Sera were inactivated at 56°C during 25 minutes, then centrifuged at 11.400 g for 30 minutes to clarify; the supernatant was stored at −20°C until assayed. Samples were analyzed for NTAb's against VEEV subtypes IAB, ID, IF, IV and VI by PRNT using Vero cells as described by Early et al. (1967) [18]. Serum samples were initially tested at a dilution of 1∶10. Those that neutralized at least 80% of inoculated viral plaque forming units (pfu) were considered positive, and in order to determine the end-point titer, they were further titrated with the same technique, using 2-fold serial dilutions.

Viruses used in this study were: a) VEEV IAB strain TC83 [19], b) VEEV ID strain 3880 -isolated for the first time in 1961 in Panama- [2], c) MDPV (VEEV subtype IF) strain 78V3531 -first isolated in 1978 in Brazil- [2], d) PIXV (VEEV subtype IV) strain BeAr35645 [20], and e) RNV (VEEV subtype VI) strain AG80-663 [9]. Viral suspensions were prepared with a 10% dilution of infected suckling mice brain in Minimum Essential Medium (MEM) 10% fetal bovine serum (FBS) and 1% antibiotics (gentamicin), centrifuged at 11.400 g for 30 minutes.

## Results

### Seroprevalence in Chaco Province

#### March-May, 2007

The 172 serum samples collected during this period were tested against VEEV subtype IAB, PIXV and RNV. These viruses were selected based on history of previous VEEV circulation in the region: detection of NTAbs against VEEV subtype I and RNV in birds, horses and rodents [21], and molecular detection of PIXV and RNV in mosquitoes [7].

Twenty three samples showed VEEV complex neutralization. Sixteen of them corresponded to inhabitants of Resistencia city and 7 to individuals of other localities of the province (Table 1).

**Table 1:** Positive samples for antibody detection against VEEV IAB, PIXV (IV) and RNV (VI) in humans from Chaco province and their titers during 2007.

		Virus				
Sample N°	VEEV-IAB	RNV	PIXV	Age^1^	Sex^2^	Place
41	−	+ (≥640)	+ (20)	20	F	Cap. Solari
43	−	+ (40)	−	3	F	R^3^
44	+ (20)	+ (320)	+ (20)	20	F	R
51	−	+ (40)	−	21	F	R
99	−	+ (160)	−	5	M	R
194	−	+ (80)	−	19	F	R
290	+ (80)	+ (2560)	+ (160)	14	M	R
295	+ (80)	+ (2560)	+ (160)	21	F	Cap. Solari
344	−	+ (160)	−	17	F	R
369	+ (80)	+ (1280)	+ (80)	21	F	R
382	+ (20)	+ (>640)	+ (80)	26	F	R
395	+ (10)	+ (320)	+ (80)	38	M	R
408	+ (10)	+ (640)	+ (80)	38	F	R
432	+ (10)	+ (>640)	−	28	M	Fontana
444	−	+ (640)	+ (10)	23	M	R
460	+ (40)	+ (640)	+ (40)	41	M	Fontana
474	−	+ (>640)	−	52	F	R
485	−	+ (80)	−	51	F	Fontana
524	+ (20)	+ (640)	+ (40)	23	F	El Sauzalito
541	+ (40)	+ (>640)	+ (20)	32	M	R
555	+ (20)	+ (320)	+ (20)	29	F	R
567	+ (20)	+ (160)	+ (20)	30	M	R
570	+ (20)	+ (>640)	+ (10)	26	F	Barranqueras

+: positive; −: negative.

1 Age (years):

2 F: female; M: male.

3 R: Resistencia.

All 23 VEEV complex reactive samples were specific to RNV; 6 of them showed monotypic reactions (Table 1). The remaining samples exhibited serological profiles compatible with heterotypical serological response, since they resulted positive to PIXV, VEEV IAB, or both of them. According to the guidelines of the Center for Disease Control and Prevention, a titer difference of four-fold or grater is used to identify the etiologic agent; when a four-fold difference is not observed, the specific etiologic agent cannot be defined [22]. Furthermore, previous studies carried out by Cámara et al. (1997) had shown cross reactions between some subtypes in one or both directions by PRNT (Table 2) [23], which should be considered in the interpretation of results.

**Table 2:** Cross reactions between VEEV IAB, VEEV ID, MDPV (IF), PIXV (IV) and RNV (VI) by PRNT (15).

	Mouse	immune	ascitic	fluid	against:
Virus/Subtype	VEEV-IAB	VEEV-ID	MDPV-IF	PIXV-IV	RNV-VI
**VEEV-IAB**	+	−	−	+/−	−
**VEEV-ID**	−	+	−	−	−
**MDPV-IF**	+/−	+/−	+	+/−	−
**PIXV-IV**	+/−	−	−	+	+/−
**RNV-VI**	−	+/−	−	+/−	+

+: Titer ≥320.

+/−: Titer between 10 and 40.

−: Titer <10.

The analysis of the results showed that titers against RNV were, in all cases, 4 times or greater than titers against PIXV, suggesting that the infecting agent had been RNV. Thus, seroprevalence for this virus in Chaco in 2007 was 14.1%. Analyzing data only from Resistencia, indicated that its seroprevalence was 10.5% in this city. Prevalence of VEEV subtype IAB and PIXV could not be calculated because of cross reactivity.

#### December 2010–May 2011

Samples collected in Pampa del Indio were tested against VEEV subtype IAB, MDPV, PIXV and RNV. Previous investigations carried out by Monath et al. (1985) [21] in Northern areas of Argentina had postulated that positive samples against subtype IAB obtained in rodents would have been due to the presence of an enzootic variety of subtype I. For this reason, in this investigation, we also included MDPV, which circulates in Southern areas of the neighbor country of Brazil.

Forty two out of the 122 samples analyzed resulted positive against some of the members of the VEE complex (Table 3). The age range of the patients was 15–88 years.

**Table 3:** Positive samples for detection of neutralizing Abs against VEEV IAB, MDPV (IF), PIXV (IV) and RNV (VI), and their titers in Pampa del Indio (Chaco Province) in the period December 2010–May 2011.

		Virus			
Sample N°	VEEV-IAB	MDPV	RNV	PIXV	Sex^1^
2	−	+ (20)	+ (20)	+ (10)	F
3	+ (20)	−	+ (80)	+ (20)	M
6	−	+ (40)	+ (80)	+ (20)	F
8	−	+ (10)	+ (20)	+ (10)	F
9	−	+ (20)	+ (40)	+ (10)	F
10	−	+ (10)	+ (80)	+ (10)	F
16	+ (20)	+ (20)	+ (40)	+ (40)	F
17	−	+ (40)	+ (40)	+ (10)	M
18	−	+ (10)	+ (80)	+ (10)	M
23	−	+ (20)	+ (20)	−	F
25	−	+ (10)	+ (40)	+ (10)	M
26	−	+ (40)	+ (160)	+ (10)	F
27	−	+ (20)	+ (20)	+ (10)	M
28	−	+ (20)	+ (160)	+ (20)	F
31	−	+ (20)	+ (20)	+ (20)	M
33	+ (20)	+ (80)	+ (160)	+ (40)	F
37	−	−	+ (40)	+ (20)	F
51	−	+ (20)	−	+ (10)	F
53	−	+ (20)	−	−	M
57	−	+ (20)	+ (20)	−	F
59	+ (10)	+ (20)	+ (80)	+ (10)	F
61	+ (10)	+ (40)	−	+ (40)	F
63	+ (10)	+ (20)	+ (80)	+ (20)	F
66	+ (10)	−	−	−	M
78	−	−	+ (40)	−	F
80	+ (80)	+ (>320)	+ (320)	+ (80)	M
81	+ (20)	+ (80)	−	+ (20)	M
85	+ (20)	+ (80)	+ (20)	−	M
86	+ (20)	+ (20)	+ (640)	+ (40)	F
87	+ (10)	+ (10)	−	+ (10)	M
90	+ (10)	+ (80)	−	+ (40)	F
93	+ (20)	+ (40)	−	−	M
94	−	−	+ (80)	−	M
95	+ (10)	+ (40)	+ (160)	−	M
99	−	−	+ (160)	−	M
104	−	−	+ (320)	−	F
109	−	+ (40)	+ (80)	+ (20)	F
110	−	−	+ (160)	−	F
112	+ (10)	+ (20)	+ (>640)	−	F
115	−	+ (20)	+ (160)	+ (20)	M
116	−	−	+ (80)	−	F
123	−	−	+ (40)	+ (10)	F

+: positive; −: negative.

1 F: female; M: male.

Most of the samples analyzed tested positive against more than one virus, suggesting the existence of cross reactivity. Only a few samples had NTAbs against one virus: sample 53 was only positive against MDPV, sample 66 only against VEEV IAB, and samples 78, 94, 99, 104, 110 and 116 only against RNV (Table 3). Among samples that tested reactive against more than one virus, a number of them presented a titer difference higher than four-fold, pointing out which was the infecting virus. Other samples did not show a four-fold titer difference, making it difficult to determine whether these individuals had been infected with one or more VEEV.

Seroprevalence of RNV was 23.0%. This value may be underestimated since the samples that could reflect double or triple infections (with titer differences lower than 4 times) were not included in this calculation.

### Seroprevalence in Corrientes Province

The 149 specimens analyzed were obtained from patients aged 0–20 years old. They were tested against VEEV subtype IAB and RNV. Two of them presented NTAbs against both RNV and TC83 strain, and only 1 tested positive against RNV (Table 4). Sample 313 corresponded to a resident of Chaco Province, for this reason, it was excluded of the analysis. The other 2 positive samples belonged to patients who lived in Corrientes city. Seroprevalence of RNV in pediatric population was 1.3%.

**Table 4:** Positive serum samples against VEEV IAB and RNV (VI) by PRNT from Corrientes Province obtained of pediatric patients (0–20 years old) between December 2006 and April 2007.

	Virus				
Sample N°	VEEV-IAB	RNV	Age (years)	Sex^1^	Place
197	−	+ (10)	8	M	Corrientes
313	+ (20)	+ (≥640)	12	F	Chaco
326	+ (10)	+ (≥640)	10	M	Corrientes

+: positive −: negative.

1 F: female; M: male.

### Seroprevalence in Salta Province

The 197 serum samples from Orán (Salta) were tested against VEEV ID, PIXV and RNV. The last two viruses were included because of previous reports about their molecular detection in other Northern regions. Subtype ID was included due to its recent detection in Bolivia (bordering country in Salta province) (Figure 1), where it has been associated with human disease with symptoms similar to dengue [6].

Five samples tested positive (age range: 21–67 years, Table 5). One specimen presented NTAbs only against RNV (sample 422); the others presented NTAbs against both RNV and PIXV, with titers varying in favor of one or the other virus. Sample 419 showed titer of NTAbs against PIXV 4 times greater, determining that this was the infecting virus; sample 619 presented a greater difference in favor of RNV (16 times). Titers obtained in samples 234 and 413 showed no significant differences (Table 5). Neutralizing antibodies against VEEV ID were not detected.

**Table 5:** Detections of VEEV ID, PIXV (IV) and RNV (VI) by PRNT in samples from Orán, Salta Province, in 2009.

		Virus			
Sample N°	RNV	PIXV	VEEV-ID	Sex^1^	Age (years)
234	+ (80)	+ (40)	−	M	67
413	+ (10)	+ (20)	−	F	32
419	+ (20)	+ (80)	−	M	48
422	+ (40)	−	−	F	21
619	+ (160)	+ (10)	−	M	49

+: positive. −: negative.

1 F: female; M: male.

## Discussion

Over the past few decades, a global resurgence of arboviruses has been detected worldwide [16]. Despite the public health relevance, the geographic range, relative impact and epidemiologic characteristics associated with arbovirus infection are poorly described in many regions of America, including Argentina. Surveillance plays an important role in the prevention of these diseases -particularly VEE-, knowing circulating viral species, susceptible population and epidemiology. Within this surveillance, studies of serological prevalence have served for a long time -and are still useful- to indicate immune status of a given population against an infectious agent, providing information of its circulation in a region. For arboviruses, PRNT is the gold standard technique due to its high sensitivity and specificity. In Argentina, there is serological evidence of the presence of VEEV since 1950's decade in humans and rodents [24], [21], [11]. Our studies detected current VEEV activity in the North part of the country.

### Chaco Province

In Chaco Province prevalence for RNV was high. Detections of this virus in individuals aged 3–5 years provide evidence of its recent circulation. Titers of NTAbs obtained in the period 2007 were, in many cases, very high (>640 and 1280), agreeing with secondary infections. In Pampa del Indio -where there are no prior registers of VEEV circulation- titers obtained against RNV showed profiles compatible with primary infections (only one sample presented titer >640). This could suggest that Pampa del Indio is an area of more recent circulation of RNV, compared to other places of Chaco province, like Resistencia city.

This is the first search of antibodies against PIXV in our country, with positive results in samples from Chaco in both studied periods. Some of these positive samples could be consequence of a serological cross reaction with RNV, while others could represent true positive results. In 2007, despite the fact that the titers obtained against RNV were four-fold higher than those obtained against PIXV and VEEV IAB, we cannot discard that, as it has been documented for some flaviviruses [22], the observed heterotypic immunological response could be the result of sequential infections by PIXV and/or VEEV IAB, especially in those samples in which the titers against PIXV were of 80 or more. For this reason, some of these individuals may have suffered infection by two or more viruses. Samples with similar titers of NTAbs against PIXV and another virus may correspond to double or triple infections, positioning Chaco province as a hyper endemic area. Pixuna virus infections are supported by previously reported molecular detections of this virus in the same region [7]. Detection of NTAbs against PIXV in Pampa del Indio represents not only another evidence of its presence in Chaco province (and Argentina), but also the wide distribution of this virus in our country.

We can assume that VEEV IAB positive specimens might be the result of antigenic cross reactivity with PIXV (in many cases titers against PIXV were 4 times greater than against VEEV IAB); however, results obtained with sample 432 in 2007 (Table 1) and sample 66 in 2011 (Table 3) could indicate activity of subtype I, since it was demonstrated that VEEV IAB does not present serological cross reactivity with RNV. This should be considered cautiously, since there is no evidence of epidemic/epizootic viral activity in our region, but the circulation of an enzootic variety of other subtype I might exist. This hypothesis was first proposed by Monath in 1985, and is supported by previous detections of antibodies against VEEV IAB in animals during the 80's [21] and in humans in 1991 [11], although no isolations of members of this subtype have been reported up to date. This leaves an open door to suspect that these antibodies previously detected could correspond to antibodies against PIXV captured by TC83 strain in the PRNT.

Many of the samples analyzed in Pampa del Indio had a positive result to MDPV. Some of them could be due to serological cross reactions with VEEV IAB and PIXV, since MDPV captures NTAbs generated against these viruses [23]. Other specimens presented titers against MDPV that were equal or higher than other viruses. These cases could represent evidence of MDPV circulation in Pampa del Indio and for the first time, in Argentina. This should be considered cautiously because, as we previously mentioned, there are no molecular detections of this or other enzootic subtype I in this country. Further studies with emphasis on the search of MDPV (or other enzootic VEEV subtype I) are needed, both by molecular detection as well as by surveillance of undifferentiated febrile human cases.

### Corrientes Province

In Corrientes, the prevalence of 1.3% for RNV in a pediatric population indicates recent circulation of the virus. The low value may show less viral activity in this city compared to Chaco (with a prevalence of 14.1% in 2009) or could be due to the fact that the detections were made in a pediatric population; this percentage may be higher in the adult or general population. The high titer of NTAbs against RNV observed in sample 326 (≥640), could indicate current infection. Our results demonstrate that VEEV strains have a silent and endemic circulation in this area and highlight the need of constant surveillance.

### Salta Province

This is the first investigation of VEEV in Salta Province. Four out of the 5 positive results exhibited serological profiles compatible with a heterotypic serological response. In two of them RNV appears to be the infecting virus, and in one PIXV. In the other two samples it was impossible to determine the etiologic agent. All positive samples were obtained from adult patients, and in consequence, it was not possible to define whether they were recent infections or not. These are the first detections of RNV and PIXV in Salta Province, showing the wide distribution of these viruses in Northern Argentina. In Orán city, some outbreaks of other arboviruses such as DENV, which share symptoms with RNV, have occurred. Previous reports in other countries of America have shown a sub-estimation of VEEV cases in regions with co-circulation with DENV or other arboviruses [4]. As this could be the case of Orán city, it is relevant to acknowledge the circulation of VEEVs in this area and in all the province, emphasizing the investigation of acute febrile cases reported as probable dengue. The fact that all the samples tested negative against VEEV ID indicates no presence of this virus in our country so far. However, since intense commercial activity is developed between Argentina and Bolivia in this area, the introduction of VEEV ID in our territory should not be discarded; therefore, surveillance should be active in this region with the aim to detect cases early.

These serological findings lead us to postulate the hypothesis that RNV is expanding into new regions, probably due to climate changes -since the climate may influence the ecology of microbial systems [25] -as well as to an increase in the commercial activities of the area. They also constitute an evidence of enzootic VEEV circulation in Northern regions of Argentina, although the role of these viruses in the production of human diseases and their impact on public health is still unknown. While detections of VEEV NTAbs in our study all belong to enzootic types, genetic studies have demonstrated that enzootic and epizootic subtypes are closely related, and a modest number of nucleotide changes can alter the viral phenotype dramatically, converting an enzootic strain to an epizootic strain [14], [26]. For this reason, it is important to perform complementary molecular studies in order to provide information about the variability of local VEEV strains.

This report is an approach to recognize which VEEV strains circulate in Northern areas of Argentina. In light of the lack of a distinctive clinical presentation and the diversity of the etiologic agents circulating in the studied area, more investigations that focus on arboviral transmission patterns, phylogenetic relationships between the strains and occurrence of clinical cases produced by RNV and a potential VEEV subtype I virus, are needed to achieve a better understanding of the impact of these viruses on human health.

## Supporting Information

Checklist S1 STROBE Checklist Manuscript PNTD-D-13-00682R1 “Venezuelan Equine Encephalitis Viruses (VEEV) in Argentina: serological evidence of human infection”.
(DOC)
